# An Insight Into the Aerosolization Pattern of Formoterol Fumarate Dry Powder Inhalation Formulation Actuated Via Two Different Inhaler Devices

**DOI:** 10.34172/apb.025.43725

**Published:** 2025-09-03

**Authors:** Leila Asadollahi, Reza Ghanbari, Soheil Abbaspour-Ravasjani, Hamed Hamishehkar, Ali Nokhodchi

**Affiliations:** ^1^Student Research Committee and Biotechnology Research Center, Tabriz University of Medical Sciences, Tabriz, Iran; ^2^Drug Applied Research Center, Tabriz University of Medical Sciences, Tabriz, Iran; ^3^Department of Materials Science and Engineering, North Carolina State University, Raleigh, USA; ^4^Research Center of New Material and Green Chemistry, Khazar University, AZ1096, Baku, Azerbaijan; ^5^School of Life Sciences, University of Sussex, Brighton, UK

**Keywords:** Aerolizer, Dry powder inhaler, Formoterol fumarate, Next generation impactor, Revolizer

## Abstract

**Purpose:**

Effective inhaled drug delivery is essential for managing bronchial asthma and chronic obstructive pulmonary disease (COPD). This study compared the aerosolization efficiency of two different dry powder inhalers (DPIs), the Aerolizer and Revolizer, using a fixed formulation of formoterol fumarate.

**Methods:**

Aerodynamic particle size distribution was measured using a next-generation impactor (NGI), and delivered dose uniformity was assessed with a dosage unit sampling apparatus (DUSA), both at a fixed flow rate of 60 L/min. Drug content was quantified using a validated high-performance liquid chromatography (HPLC) method, and performance metrics were analyzed using CITDAS software. Data were averaged (mean±SD) and compared by statistical tests (e.g., ANOVA or t-tests), with *P*<0.05 indicating significance.

**Results:**

The Aerolizer achieved a fine particle dose (FPD) of 4.71 µg, which was 2.39 times higher than that of the Revolizer (1.97 µg). It also delivered approximately 20% greater overall dose and showed more consistent deposition in the NGI stages. While both devices demonstrated similar fine particle fractions (FPFS), the difference in FPD was primarily due to the higher emitted dose from the Aerolizer. The use of a fixed flow rate allowed direct comparison of device performance.

**Conclusion:**

These findings highlight the significant influence of device design on DPI performance, even when the formulation remains constant. The Aerolizer, a low-resistance inhaler, showed superior delivery efficiency than the Revolizer under standardized conditions. Future studies should include pressure-drop–adjusted or patient-simulated testing to better reflect clinical inhalation profiles and further explore how device mechanics influence drug delivery.

## Introduction

 Chronic obstructive pulmonary disease (COPD) and asthma are two of the most common respiratory conditions worldwide. According to the World Health Organization (WHO), COPD was the fourth leading cause of death globally in 2021, responsible for 3.5 million deaths, mostly in low- and middle-income countries with high exposure to tobacco smoke and household air pollution. Asthma, meanwhile, affected an estimated 262 million people in 2019 and caused over 450,000 deaths, particularly in regions with limited access to proper diagnosis and treatment. Although both conditions are incurable, inhaled medications remain essential for long-term symptom management and quality of life improvement.^1,2^ Therefore, optimizing pulmonary drug delivery, especially through dry powder inhalers (DPIs), is vital for achieving effective and consistent treatment outcomes.

 DPIs are widely used to deliver medications to the lungs via breath-actuated dispersion of micronized powder, providing both local and systemic effects.^3^ Their effectiveness relies not only on the stability and characteristics of the powder formulation, but also device’s internal design and the patient’s inhalation technique.^4^ For example, capsule-based DPIs require adequate user dexterity and a fast inhalation to disperse the powder.^5^ Notably, many commercial DPIs exhibit relatively low efficiency: reported fine-particle fractions (percentage of the emitted dose < 5 µm) are often only 20–30% at a flow rate of 60 L/min.^6^ In this study, we used formoterol fumarate, a long-acting β₂-agonist widely prescribed for asthma and COPD maintenance therapy, as the test drug to evaluate and compare device performance.^7,8^

 Since the emergence of the COVID-19 pandemic, inhalation therapy has received renewed attention, with clinicians emphasizing the continued use of controller medications and reports noting a 14.5% relative increase in adherence to asthma and COPD inhalers during the first lockdown, reflecting heightened patient awareness of respiratory health risks.^9,10^ In response, many pharmaceutical companies have shifted toward producing unit-dose inhalation capsules without a paired device, leaving patients or providers to select an appropriate inhaler. This may lead to variability in treatment outcomes, as device performance depends on design, powder formulation, and patient factors such as age and inspiratory flow, highlighting the importance of regulatory oversight and consideration of device reliability alongside innovation.^11^ This study compares two commercially available capsule-based DPIs, the low-resistance Aerolizer and the medium-resistance Revolizer, under controlled conditions.^12,13^ Aerodynamic particle size distribution and delivered dose were measured at a standardized pharmacopeial flow of 60 L/min, which is in the mid-range of flows achievable by most patients and coincides with prior findings that the Aerolizer’s aerosolization is optimized near 65 L/min, and the Revolizer likewise achieves high lung deposition around 60 L/min.^6,14,15^ Unlike many earlier studies that varied both device and formulation or tested each inhaler at different flow rates, our experimental design holds the formulation and flow rate constant for both DPIs. This novel approach allows a direct comparison of how device architecture, such as airflow pathways, capsule chamber geometry, and deaggregation mechanism, independently affects aerosolization performance. The results are intended to inform future DPI design and regulatory evaluation, and to support evidence-based selection of inhalers in clinical practice.

## Materials and methods

###  Materials

 Formoterol fumarate DPIs and Aerolizer (including batch numbers: BVC23) utilized in this study were obtained from Novartis Pharma Stein AG, Basel, Switzerland. Each capsule used in the study was a Foradil^®^ (Novartis) unit-dose DPI capsule containing 12 µg of micronized pharmaceutical-grade formoterol fumarate blended with inhalation-grade lactose monohydrate as the carrier. The total capsule fill weight is approximately 25 mg. The Revolizer was sourced from Cipla, Mumbai, India. Type A/E-1 glass fiber filter paper was purchased from Pall Life Sciences, Germany. All solvents and chemicals used in the study were of HPLC grade and obtained from Dr. Mojallali’s company, Tehran, Iran. Additional chemicals were procured from Merck & Co., Inc., New York, USA.

## Methods

###  Characteristics of devices

**Figure 1:**
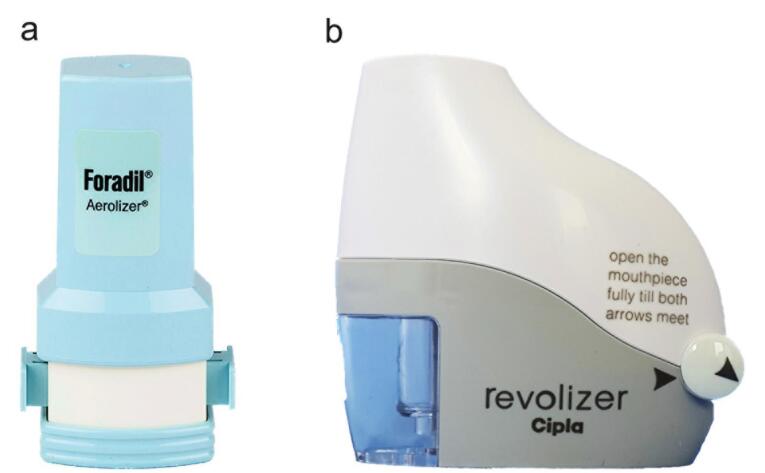
Comparison of inhaler devices (a) Aerolizer, (b) Revolizer

Figure 1 provides detailed descriptions of the two employed devices.

####  Assessment of fine particles using next-generation impactor and delivered dose uniformity with dosage unit sampling apparatus (DUSA)

 The aerodynamic particle size distribution of formoterol fumarate delivered via Aerolizer and Revolizer was evaluated using a Next-Generation Impactor (NGI, Copley Scientific, UK) with a USP induction port and pre-separator. Testing was performed at a calibrated flow rate of 60 L/min, as specified in US*P*< 601 > , using a critical flow controller (TPK 2000) and a vacuum pump (HCP5). The pressure drop measured across the device was close to 4 kPa. The actuation duration was set to 4 seconds to deliver 4 L of air. Ten capsules were tested per run, with each run repeated three times for both inhalers. A fixed flow rate of 60 L/min was applied to both devices. Before each run, NGI cups were coated with 1% (w/v) Tween 80 and dried. After actuation, the USP induction port, pre-separator, NGI stages 1–7, and the micro-orifice collector (MOC) were dismantled and washed using a solvent system consistent with the HPLC mobile phase: acetonitrile and phosphate buffer (30 mM sodium dihydrogen phosphate monohydrate, 3.5 mM phosphoric acid, pH 3.1 ± 0.1). Washing volumes were as follows: 10 mL for the capsule and induction port, 35 mL for the pre-separator, and 4 mL for each NGI stage and the MOC. The drug content in each wash was quantified using the validated HPLC assay described in Section 2.2.3.^16^

 To examine the uniformity of the delivered dose of formoterol, a DUSA apparatus (Copley Scientific, UK) was employed. For analysis, a 47-mm diameter Type A/E−1 glass fiber filter paper from Pall Life Sciences, Germany, was placed between the sample collection tube and the filter-support base. In each assessment, one capsule was tested with 10 repetitions at a constant flow rate of 60 L/min using the Aerolizer and Revolizer devices within the DUSA apparatus. After actuation, the DUSA assembly was dismantled, and all components were washed with 10 mL of solvent to ensure complete recovery of the drug content. The amount of formoterol remaining in different parts was then evaluated and measured using the HPLC system.^17^

 For DUSA testing, the airflow duration was set to 2 seconds (equivalent to 2 L of air at 60 L/min), as per US*P*< 601 > requirements for delivered dose uniformity assessment.

 Data Analysis was conducted using Copley Inhaler Testing Data Analysis Software (CITDAS, version 3.10), which calculated parameters such as fine particle fraction (FPF), mass median aerodynamic diameter (MMAD), and geometric standard deviation (GSD). MMAD represents the particle size at which 50% of the aerosol mass is smaller and 50% is larger.^18^ FPF indicates the proportion of the emitted dose represented by drug particles with an aerodynamic diameter smaller than 5 μm.^19^

####  Formoterol fumarate HPLC assay and homogeneity

 The amount of formoterol fumarate was measured using a validated high-performance liquid chromatography (HPLC) method according to the European Pharmacopoeia 6.0. Separation was performed using an HPLC system (1260 Infinity II LC System, Agilent Technologies, Santa Clara, CA) on an octadecyl silyl silica gel C18 column (25 cm × 4.6 mm, 5 µm). The column temperature was maintained at 25 °C, with a flow rate of 1 ml/min and an injection volume of 20 μl. The mobile phase consisted of acetonitrile (Phase A) and a buffer solution of 30 mM sodium dihydrogen phosphate monohydrate and 3.5 mM phosphoric acid, pH adjusted to 3.1 ± 0.1 (Phase B).

 Gradient elution method was used in the HPLC procedure. Initially, 16% phase A and 84% phase B were employed followed by transitioning to 70% phase A and 30% phase B from 10 to 37 minutes, and returning to 16% phase A and 84% phase B from 37 to 40 minutes. The solvents were held constant from 40 to 55 minutes, followed by column washing and reconditioning.

 Chromatograms were obtained at 214 nm, where formoterol fumarate exhibits maximum absorbance. Compound identification relied on comparing retention time and UV spectra (200 to 400 nm) with the standard. Quantification was performed using standard curves with regression coefficients (R2) ≥ 0.99. Data analysis was conducted using Chrom Gate Client/Server, version 3.1.7, to calculate the area under the curve for the formoterol fumarate peak.

####  Statistical analysis

 Each experiment was replicated three times, and the resulting data were analyzed using GraphPad Prism 9.0.2 software (GraphPad Software, San Diego, CA). The independent two-way analysis of variance (ANOVA) variance test was used with multiple comparisons between data using the LSD significant difference test. A p-value less than 0.05 was considered statistically significant. The ANOVA as well as the designed general linear model for the logarithmically transformed data (concentration) obtained from DUSA were carried out according to drug analysis guidance documents (USA, Canada).^20^ All statistical analyses for DUSA were performed using SPSS 16. After obtaining the SPSS tables, to compare and analyze the data, the geometric mean ratio (GMR), the ratio estimate, and inter- and intra-capsule coefficient variation parameters were calculated using the following formulas:


$$The\;geometric\;mean\;ratio\;\left(\%\right)=\frac{geometric\;mean\;\;Revolizer}{geometric\;mean\;\;Aerolizer}*100$$

$$\text{Ratio}\;\text{estimate }\left(\text{\%}\right)=100\;e^{\;\left(\text{geometric}\;\text{mean}\;\text{Revolizer }-\text{ geometric mean}\;\text{Aerolizer}\right)\;}$$

$$\text{Inter capsule coefficient variation }=100\;\sqrt{e{\;}^{\text{Mean Square Inter Capsules}}-1}$$

$$\text{Intra capsule coefficient variation }=100\;\sqrt{e{\;}^{\text{Mean Square Error }}-1}$$

## Results and Discussion

**Figure 2:**
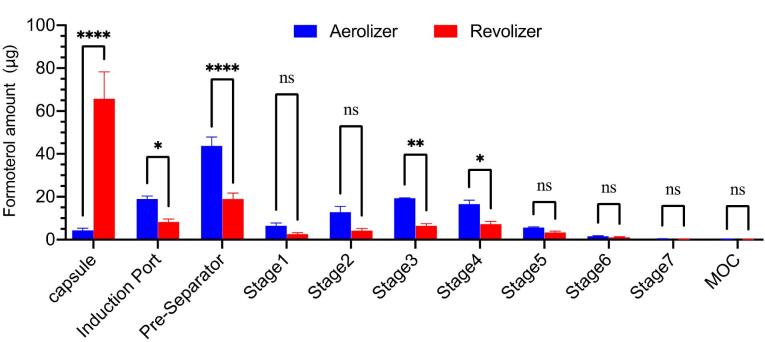
Formoterol deposition in the next-generation impactor using Aerolizer and Revolizer. Data presented as mean ± SD (n = 3). ns: not significant, *P < 0.1, ** P < 0.01, *** P < 0.001, and **** P < 0.0001 compared to multiple comparisons

Figure 2 shows the aerodynamic particle size distribution of formoterol fumarate from the Aerolizer and Revolizer at a flow rate of 60 L/min, based on ten capsules per run. Both inhalers exhibited distinct size distribution profiles across NGI stages, indicating device-dependent differences in aerosolization.

 The Revolizer retained 15.16-fold more drug in the capsule after actuation compared to the Aerolizer, suggesting reduced emission efficiency likely due to capsule motion or airflow dynamics. In contrast, the Aerolizer released more drug but also retained approximately 2.3 times more in the pre-separator, possibly reflecting partial aggregation or incomplete deagglomeration. Despite this pre-separator loss, the Aerolizer achieved a significantly higher fine particle dose (FPD), 4.71 µg compared with 1.97 µg for the Revolizer (*P* < 0.05), and total delivered dose at downstream impactor stages. The FPF was similar for both devices at approximately 37%. This similarity arose because both emitted dose and FPD were low in the Revolizer, while both were substantially higher in the Aerolizer, indicating more efficient dispersion. Overall, the Aerolizer showed greater deposition at every impactor stage, with statistically significant improvements in both FPD and delivered dose (Table 1). Although high standard deviations were observed for some parameters, such variability is expected in capsule-based DPIs due to factors such as piercing characteristics, airflow resistance, and turbulence.^21^

**Table 1:** Key data from the NGI analysis of formoterol with both Aerolizer and Revolizer

**Device**	**FPD**^a^ ** (µg)**	**FPF**^b^ ** (%)**	**MMAD**^c^ ** (µm)**	**GSD**^d^	**Average of the mean delivered dose (%)**
Aerolizer	4.71 ± 0.32	37.41 ± 1	3.32 ± 0.06	1.96 ± 0.06	98.51 ± 25.24
Revolizer	1.97 ± 0.31^e^	37.33 ± 4.99 ^ns^	2.93 ± 0.17 ^ns^	2.16 ± 0.14 ^ns^	78.85 ± 29.98^f^

a: Fine particle dose; b: Fine particle fraction; c: Mass median aerodynamic diameter; d: Geometric standard deviation. Note: The results were calculated as the mean ± standard deviation (n = 3). ns: not significant, ^e^*P* = 0.0024, and ^f^* P* = 0.049, as compared Aerolizer.

 Our experiments confirmed that device design critically affects DPI performance.^22^ According to previous studies, particles around 5 µm deposit mainly in the upper airways, 2–5 µm in central airways, and 0.5–2 µm in peripheral lung regions.^23^ Both the Aerolizer and Revolizer produced aerosol particles within the ideal MMAD range of 1–5 µm, necessary for deep-lung deposition.^24^ The Aerolizer generated an MMAD of 3.32 µm and the Revolizer 2.93 µm, both within the target range. However, the particle size distribution was more uniform for the Aerolizer, with a GSD of 1.96 compared to 2.16 for the Revolizer. A GSD below 2 indicates a narrower size distribution, suggesting that the Aerolizer could provide more consistent deposition and better therapeutic uniformity.^25^

 The delivered dose differed substantially between devices. The Aerolizer reached 98.51% of the label claim, compared to 78.85% for the Revolizer under identical testing conditions. The statistical analysis of DUSA data was performed using a general linear model, and results were reported in three key metrics: (A) the significance value and 90% confidence interval (CI) for the parameter being analyzed; (B) the GMR and ratio estimate (%) of mean concentrations between the Aerolizer and Revolizer; and (C) the inter- and intra-capsule coefficient of variation (CV) for each device. Comparative results are presented in Table 2 and Table 3, which also include within-capsule variance estimates, mean square error for CI calculation, and significance levels.

**Table 2:** The ANOVA for the design model for the dependent variable of Ln concentration from DUSA

**Source**	**Sum of Squares**	**df**^a^	**Mean Square**	**F**^b^	**Significant**^c^
Corrected Model	1.284	10	0.128	2.316	0.111
Intercept	107.512	1	107.512	1938.989	0.000
DPIs	0.285	1	0.285	5.144	0.049
Capsules	0.999	9	0.111	2.001	0.158
Error	0.499	9	0.055		
Total	109.295	20			
Corrected Total	1.783	19			
Inter Capsules			0.027		

a. Variable degrees of freedom; b. Fisher–Snedecor parameter; c. Significant less than 0.05 was considered statistically significant.

**Table 3:** The geometric mean ratio (GMR), ratio estimate (%) of the geometric mean of Ln concentration, their lower and upper 90% confidence intervals, and the inter- and intra-capsule variations derived from Table 2

**Ln concentration**	**Geometric**		**Arithmetic**		**GMR (%)**	**Ratio Estimate (%)**	***P*** **value**^b^	**90% CI**^c^		**Capsule CV**^d^ ** (%)**	
	**Mean**	**SD**^a^	**Mean**	**SD**				**Lower**	**Upper**	**Intra**	**Inter**
Revolizer	2.19	0.30	9.46	3.41	90.12	78.66	0.049	64.93	95.52	23.87	16.77
Aerolizer	2.43	0.27	11.82	2.87							

a. Standard. deviation; b. *P* valuesless than 0.05 was considered statistically significant; c. Confidence interval; d. Coefficient variation.

 Given the non-normal distribution of concentration data in biological studies, geometric statistics were used instead of arithmetic values.^26^ Accordingly, concentrations were log-transformed, and the resulting mean and standard deviation of Ln concentrations were converted into geometric means (GMs) and GSDs. The model showed that device type had a statistically significant effect on delivered dose (*P* = 0.049), while capsule substitution did not (*P* = 0.158), indicating that swapping capsules between devices did not influence the outcome. The GMR of Aerolizer to Revolizer concentrations was 90%, corresponding to a ratio estimate of 78.66%, indicating lower delivered concentrations from the Revolizer, and the 90% confidence interval (64.93%–95.52%) fell outside the pre-specified equivalence range (80%–125%), confirming a significant non-equivalence in aerosol delivery. Within- and between-capsule coefficients of variation were both under 30%, demonstrating acceptable reproducibility. Overall, the Aerolizer consistently outperformed the Revolizer, delivering a higher FPD with less variability under standardized 60 L/min flow conditions.^20^

 Our principal finding is that inhaler internal design, not airflow resistance alone, significantly influences aerosol performance. Despite being a low-resistance device, the Aerolizer delivered a larger FPD than the medium-resistance Revolizer. This may seem counterintuitive, as higher-resistance DPIs are often associated with more efficient powder deagglomeration at lower flow rates.^25,27^ However, the Aerolizer’s superior performance can be explained by its optimized internal architecture, including airflow pathways and capsule dispersion chamber geometry, which likely enhanced turbulence and powder deagglomeration.^28^ In contrast, the Revolizer’s internal airflow structure resulted in greater powder retention and a lower delivered dose, despite its higher resistance. Quantitatively, the Aerolizer’s delivered dose exceeded the Revolizer’s by approximately 20%, and the 90% confidence interval confirmed significant non-equivalence in performance.

 Although the effect size for device type was modest, the GMR of 90% suggests improvement in drug delivery, which may have clinical relevance, especially for patients with limited inspiratory capacity. These results align with prior literature emphasizing that patient-generated pressure drop, rather than peak flow rate alone, is a key driver of DPI performance. A pressure drop of ≥ 1 kPa is generally sufficient for effective lung delivery.^29^ So, both the Revolizer and the Aerolizer achieved efficient aerosolization under the standardized 60 L/min flow used in this study. Previous investigations also demonstrate that small structural changes, such as air inlet size, mouthpiece length, and grid geometry, can significantly alter FPF and drug retention.^22^ These observations support our finding that subtle differences in device architecture have measurable effects on aerosol output. Computational fluid dynamics studies further confirm that turbulence patterns inside inhalers strongly influence powder dispersion and deposition, providing additional insight into airflow optimization.^30,31^

 While this study evaluated only two devices, both were selected for clinical relevance and differing resistance profiles. Using identical capsule formulations and a fixed flow rate eliminated confounding variables related to formulation or patient effort, allowing the isolated assessment of device architecture. Inter- and intra-capsule variability remained low (< 30%), indicating reliable and reproducible results. Nevertheless, real-world inhalation profiles vary with patient effort and lung function, particularly in severe asthma or COPD. Future studies incorporating patient-simulated flows, pressure-drop–adjusted conditions, and a broader range of DPI designs would provide more generalizable insights.

## Conclusion

 In conclusion, this study demonstrates that inhaler design significantly influences the performance of DPIs, independent of formulation. Both the Aerolizer and Revolizer generated aerosols within the optimal aerodynamic size range for lung deposition, but the Aerolizer consistently achieved a higher FPD and overall delivered dose, reflecting superior deposition efficiency. These findings highlight that low-resistance devices with optimized internal airflow geometry and dispersion mechanisms may offer clinical advantages, particularly for patients with limited inspiratory capacity, such as those with asthma or COPD. For clinicians and regulators, this underscores the importance of considering not only airflow resistance but also internal device architecture when selecting or approving inhalers. Expanding future research to include a wider range of devices, patient inhalation profiles, and pressure-drop–adjusted testing will help refine inhaler selection strategies and ultimately improve therapeutic outcomes in chronic respiratory disease management.

## Competing Interests

 The team of authors of this article declares that they don’t have competing interests that could potentially influence or bias the results of this research.

## Data Availability Statement

 The team of authors is committed to ensuring that the data supporting our findings in this research will be made available to interested parties upon reasonable request. The requested data are available by contacting the corresponding authors.

## Ethical Approval

 This study was approved by the Ethics Committee of Tabriz University of Medical Sciences, Tabriz, Iran (Approval code: IR.TBZMED.VCR.REC.1402.279).
